# Green credit policy and residents’ health: quasi-natural experimental evidence from China

**DOI:** 10.3389/fpubh.2024.1397450

**Published:** 2024-07-04

**Authors:** Mengyu Wang, Yichun Wang, Bingnan Guo

**Affiliations:** School of Humanities and Social Sciences, Jiangsu University of Science and Technology, Zhenjiang, China

**Keywords:** green credit, residents’ health, difference-in-differences model, environmental pollution, China

## Abstract

**Background:**

Residents’ health plays an important role in economic prosperity and national development.

**Methods:**

The research analyzes data from 262 prefecture-level cities in China spanning the period from 2010 to 2021. Utilizing the implementation of green credit policy in China as a quasi-natural experiment, the paper employs the time-varying Differences-in- Differences (DID) model to evaluate the influence of green credit policy on residents’ health.

**Results:**

The paper results show that: (1) the green credit policy significantly improves residents’ health, and this conclusion still holds after a series of robustness tests. (2) Mechanism analysis reveals that the green credit policy affects residents’ health through the improvements of the environment and the elevation of public services standards in demonstration cities. (3) Heterogeneity analysis shows that the impact of green credit policy on residents’ health is more significant in the western cities and resource-based cities than in the central-eastern cities and non-resource-based cities. This paper explains the specific path and realization of green credit policy to enhance residents’ health, which provides a reference for further designing and improving effective green credit policy.

**Discussion:**

The deficiencies within the green credit policy has resulted in limited improvements. It is recommended that China should broaden the ambit of the green credit policy and refine the criteria for its execution.

## 1 Introduction

As a crucial component of human capital, health offers a key support in enhancing socioeconomic development. Despite continuous advancements in China’s economic growth and social living standards, the overall health status of its population has a darkening outlook (1). Report on *Chinese Residents’ Chronic Diseases and Nutrition (2023)* (2) reveals a prevalence of medical conditions among adults: hypertension (27.5%), diabetes mellitus (11.9%), and hypercholesterolemia (8.2%). Moreover, the prevalence of chronic obstructive pulmonary disease among individuals aged 40 and older is alarmingly high (13.6%). Specifically, all of these figures have witnessed an upward trend compared to the data released in 2019. In response to these challenges, the Chinese government has implemented an array of policies aimed at enhancing the health status of its citizens. *The “Healthy China 2030” Planning Outline*, issued in December 2016, sets the target of significantly enhancing health service capacity by the year of 2030. In addition, *the Healthy China Action (2019–2030)*, promulgated by the Healthy China Promotion Committee in July 2019, actively promotes healthy lifestyles and behaviors among residents to cultivate a society characterized by reduced morbidity and improved well-being. Through the joint efforts of various stakeholders of society, the national health literacy rate has steadily risen from 9.48% before 2013 to 27.8% in 2023.

Recognizing the critical importance of environmental sustainability, the Chinese government has devised a major strategic plan stressing the importance of “promoting green development and harmonious coexistence between nature and population.” In general, the burgeoning field of green finance has become an indispensable tool for advancing these objectives. By offering financial services for project investment, operation, and risk management in sectors such as environmental protection, energy conservation, clean energy, green transportation, and sustainable infrastructure, green finance accelerates the transition toward a low-carbon economy. Cultivating the healthy development of green finance is crucial for facilitating the growth of a green economy and environmental remediation, thus leading to significant improvements in both the quality and quantity of economic development. Furthermore, green finance holds potential for achieving a win-win scenario for both economic prosperity and environmental well-being. Therefore, green finance not only serves as a driver of economic growth but also constitutes an indispensable component in protecting public health. The central government reports prominently feature “*Healthy China*” as an integral component of the country’s overarching development objectives for modern construction. The report advocates prioritizing public health, enhancing health promotion policies, advancing the construction of a Healthy China, and effectively protecting the well-being of the populace. Addressing the burgeoning public health needs necessitates the exploration of novel pathways (3). In light of increasing environmental challenges, the burgeoning field of green finance offers significant support for capital requirements and optimal resource allocation in relevant sectors. This evidently presents a significant opportunity to effectively minimize the negative effects of environmental pollution on public health. However, despite the widespread recognition of green finance’s potential in environmental protection and sustainable development, research concerning its effect on public health remains relatively scarce. In this context, conducting comprehensive research into the effects of green finance development on residents’ health and its underlying mechanisms in China can offer valuable insights. Such research can inform government policymaking in green finance, thereby promoting green economic development and the construction of a Healthy China. Moreover, it can facilitate the realization of the national strategy for the coordinated development of population health, the economy, and society (4).

Therefore, compared with the existing research, this paper’s marginal contributions are mainly in three aspects. First, this paper explores the impact of green credit policy on residents’ health from the perspective of environmental improvement and public services enhancement. Existing studies mainly focus on the impact of green credit policy on corporate environmental technology and industrial green transformation. However, there are fewer papers on the impact of green credit policy on residents’ health through environmental improvement and public service enhancement. Through the improvement of environmental quality, the health of residents is in line with the general equilibrium theory of health, verifying the mediating role played by the environment. Second, this paper adopts the difference-in-differences method (DID) to test the effect of green credit policy on residents’ health under the influence of different factors. The difference-in-differences approach reduces the issue of endogeneity significantly, leveraging both grouping variables and policy dummy variables in regression analysis to determine the effect of policy with greater reliability. Third, this paper discusses the aspect of heterogeneity. The effect of the green credit policy on residents’ health demonstrates significant differences across urban regions and resource endowments. These findings offer robust support for the promulgation of green policies and regulations by governmental and financial institutions.

**Figure 1:**
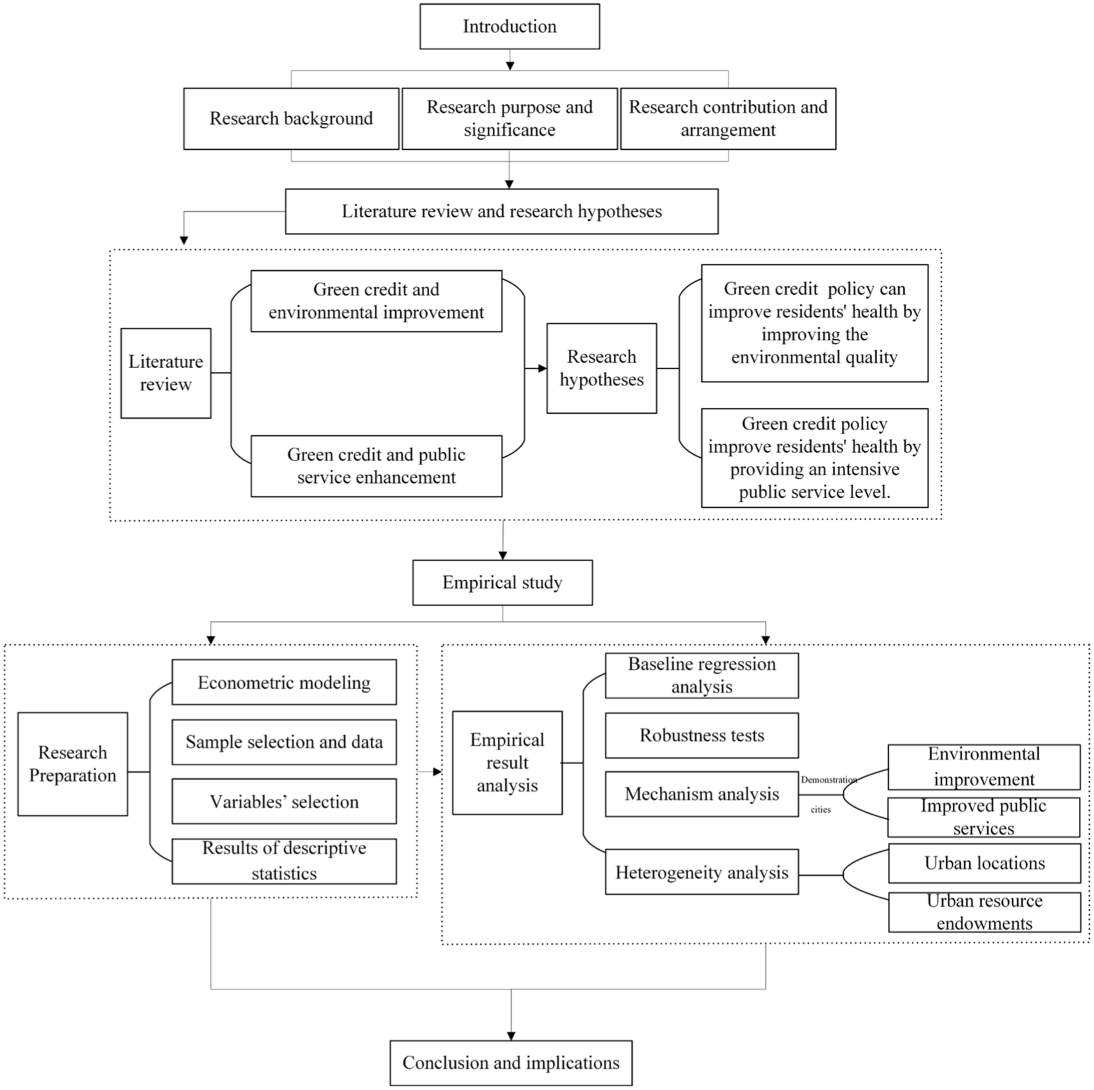
Research flow chart.

The rest of the paper is organized as follows. The second section contains the relevant literature review and research hypotheses. The third section presents the data sources, variable definitions, and descriptive statistics. The fourth section reports the research design, baseline regression results, and robustness test. The fifth section explores the specific mechanism in demonstration cities. The sixth section conducts a further study on the heterogeneity of the green credit policy. The last one, there are conclusions and policy recommendations (Figure 1).

## 2 Literature review and research hypotheses

The green credit policy aims to improve residents’ health by improving environmental quality and enhancing public services. On one hand, the green credit policy can enhance industrial structures and directly enhance urban environmental quality, it will contribute to the improvement of public health. On the other hand, this policy addresses ecological issues from the perspective of financial and fiscal integration, effectively alleviating the fiscal pressure on local governments. It offers increased fiscal support for local public infrastructure, enhancing the level of public services and indirectly improving public health. Therefore, this article reviews literature regarding both green credit and environmental enhancement, as well as the improvement of public services.

Scholars have conducted more studies on the implementation of green credit policy in China. Zhou and Long (5) examined balanced panel data from 31 provinces in China, which showed that green credit can significantly promote the performance of ecosystem governance in different provinces. Guo et al. (6) selected macro panel data of 30 provinces (cities) in China from 2000 to 2019 for standardized tests, and concluded that green finance can significantly reduce carbon emissions from agriculture. Li et al. (7) argued that the development of green credit in the Yellow River Basin can effectively decrease the local carbon emission intensity and promote low-carbon transition. Li et al. (8) found that green credit can increase industrial green total factor productivity by changing the structure of energy consumption. Xu and Zhu (9) showed that China’s overall green governance index and green financial policies resulted in a significant decrease in environmental pollution. Xia et al. (10) presented evidence that green finance significantly cultivates green innovation and benefits the promotion of green innovation both in local jurisdictions and adjacent provinces. Guo et al. (11) contended that the effect of executing new energy demonstration cities is more significant in central and western regions, as well as in smaller and mid-sized urban areas, particularly regarding the emission reduction effect. In addition, the extent of environmental information disclosure is influenced by regional factors, industrial pollution types, and the characteristics of the business (12).

The consensus in the existing articles is that the green credit policy exerts a positive effect on environmental improvement. Wang et al. (13) concluded that green finance has a negative impact on carbon emissions in BRICS countries. Hailiang et al. (14) had also found that green finance, renewable energy consumption, and technological innovations performed well in protecting the environment by reducing carbon emissions. Zhang et al. (15) took 49 countries that issued green bonds as a sample and concluded that green finance effectively mitigates environmental pollution and climate change. The impact of environmental problems on residents’ health can be traced back to Grossman (16) who included environmental factors in the health production function. Since then, numerous studies have confirmed that environmental pollution is an important factor influencing residents’ health. According to existing studies, the aggravation of environmental pollution can increase the infant mortality rate (17), raise the probability of childhood obesity and other diseases (18) and the probability of cardiovascular disease and lung cancer in the population (19) as well as the level of mental depression in the older adult (20). Some studies have even found higher mortality rates among COVID-19 infected individuals in areas with higher PM_2.5_ concentrations (21). Ahn et al. (22) concluded that prolonged working hours and work-related stress can deteriorate the residents’ health. Raynor et al. (23) identified a significantly higher incidence of mental health deterioration in people exposed to COVID-19 shocks. Aeschbach et al. (24) selected an active control group for a longitudinal randomized controlled trial and concluded that a customized MBP may improve some aspects of residents’ positive mental health. It is acknowledged that green credit is critical in curtailing pollution and energy consumption among high-emission enterprises (25). In conclusion, this paper proposes:

*Hypothesis 1*: Green credit policy can improve residents’ health by improving the environmental quality.

A comprehensive review of literature indicates that public services exert a significant effect on the health of individuals. Research by Bleakley and Ellis (26) highlighted the critical role of public health policy research in shaping the well-being of adolescents, both physically and mentally. Similarly, Lai et al. (27) explored how a supportive community environment is instrumental in enhancing the physical and mental health of older adults. Further analysis by Elliott et al. (28) indicated a correlation between community cohesion and the subjective well-being of its members. A robust sense of community cohesion is associated with improved well-being among residents. This is partly due to the tangible support networks in communities that offer financial aid, nursing, and transportation services, which alleviate mental and physical burdens and offer a safety net for residents. In environmental economics, Putnam, Hu et al. (29) presented an argument that green credit has a significant effect on the evolution and advancement of China’s industrial framework. Zhu (30) delved deeper into this relationship by integrating concepts of green credit, technological innovation, industrial structure enhancement, and the health of residents. This analysis theorizes and evaluates the effect of green credit and technological innovation on both industrial structure enhancement and the health of the populace. The findings suggest that the application of green credit policy can amplify regional public services, thereby enhancing the health of residents. Hou et al. (31) proposed that urban public services and ecosystem services can achieve positive synergies through the decentralization of public services distribution. Conversely, Bo et al. (32) identified that inefficient public expenditure and urban densification are contributing factors to a host of economic and environmental challenges. Mitchell and Popham (33) discussed the association between the availability and quality of green spaces and the health of residents, noting that while quality green spaces are beneficial, those of poor quality may actually be harmful to public health. Besides, Hu et al. (34) argued that the policy on Energy-consuming rights trading has a significant effect on the quality of urban innovation, especially in cities that are resource-based or of a larger scale, suggesting that while well-maintained green spaces are associated with health benefits, those of inferior quality may be counterproductive to public health objectives. In summary, this paper proposes:

*Hypothesis 2*: Green credit policy improves residents’ health by providing an intensive public services level.

## 3 Model design and variable data description

### 3.1 Econometric modeling

Intending to test the previous research hypotheses, this paper refers to the previous literature (12, 35) and uses the green credit policy as a quasi-natural experiment and adopts the time-varying DID model to conduct the test. Setting the econometric model as:


(1) $$Rod_{\mathrm{it}}=\alpha +\beta \times DID_{\mathrm{it}}+\mathrm{\gamma Control}s_{\mathrm{it}}+\mu_{i}+\lambda_{t}+\upsilon_{j}+\varepsilon_{\mathrm{it}}$$

Where $Rod_{\mathrm{it}}$ is the explained variable, representing residents’ health. $DID_{\mathrm{it}}$ is the explanatory variable for the green credit policy and takes the value of 1 for the year in which the green credit policy was implemented and thereafter, otherwise it takes the value of 0. Controls represents the control variables. $\mu_{i},\lambda_{t}\;\mathrm{and}\;\upsilon_{j}$ represent the time-fixed and city-fixed effects and individual-fixed effects, respectively. And $\varepsilon_{\mathrm{it}}$ represents the random error term. β represents core regression coefficients.

### 3.2 Sample selection and data

This research examines the effect of green credit policy implementation on residents’ health. Leveraging the promulgation of green credit policy across different regions as a quasi-natural experiment, this research analyzes data from 262 prefecture-level cities in China spanning the period from 2010 to 2021. The year 2012, which marks the issuance of the *Green Credit Guidelines* by the Chinese government, serves as the starting point for policy implementation. Following the approach of Wang et al. (36), missing data for key variables were excluded, resulting in a final sample of 3,144 observations, comprising 158 cities designated as treatment cities for green credit policy.

**Figure 2:**
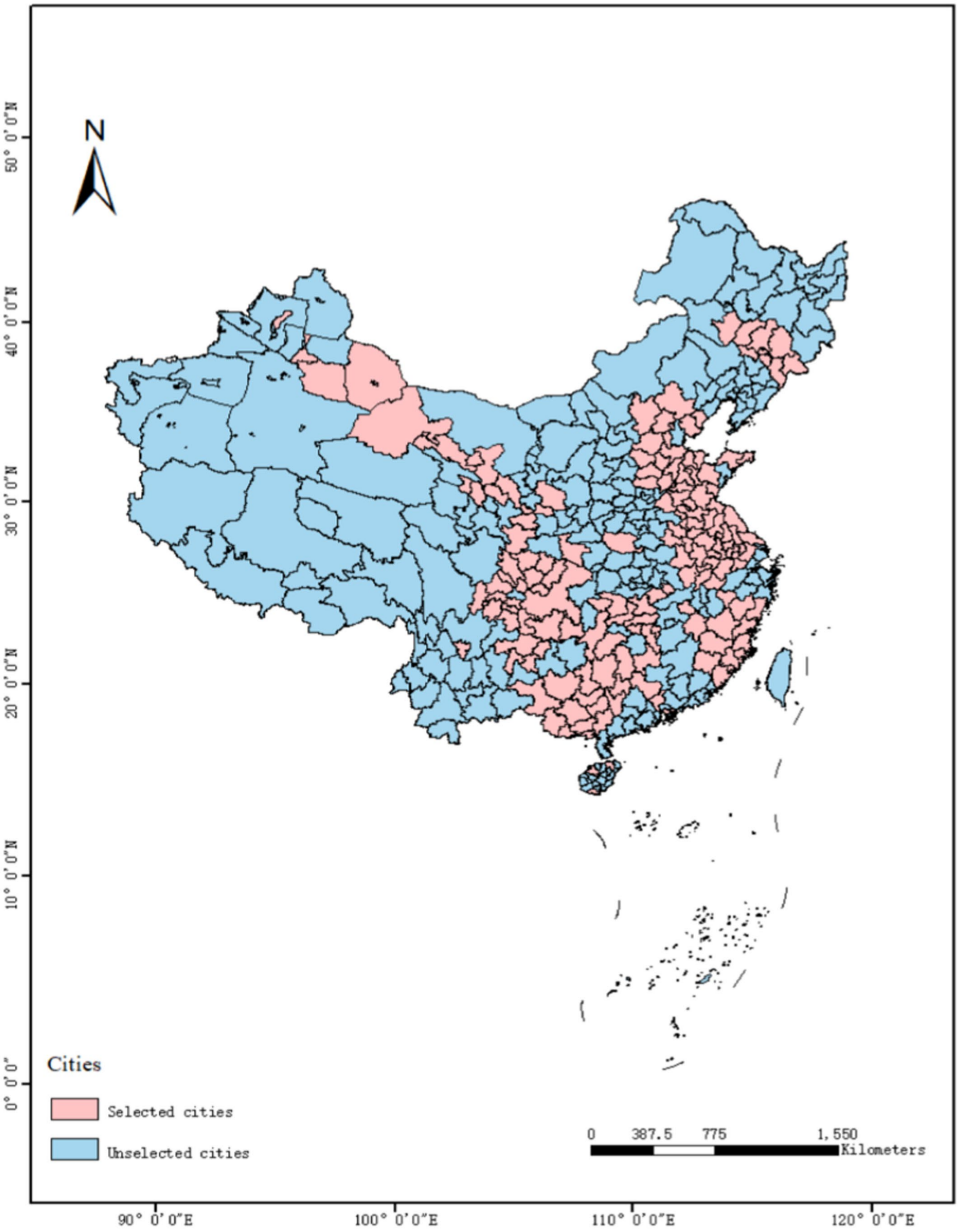
The distribution of demonstration cities.

All data in this paper are obtained from the *China Urban Statistical Yearbook*, *China Industrial Statistical Yearbook*, etc., and the existence of local missing data is supplemented by multiple interpolation methods (Figure 2).

### 3.3 Variables’ selection

#### 3.3.1 Explained variable

The explained variable in the research is residents’ health (*rod*). The usual way of measuring the health of all residents’ health in an area is to use indicators such as mortality rates, average life expectancy, and respondents’ subjective perception of their health. The “*Healthy China 2023”* program outlines mortality as the primary health indicator, so this research uses mortality rates by region as a measure of residents’ health.

#### 3.3.2 Explanatory variable

*DID* is the interaction term between the region dummy variable and the time dummy variable, which is 1 if the area involved in the policy after its enactment and 0 if not, where the time dummy variable is 1 for the years in 2012 and after that, and 0 for the years before 2012. The region dummy variable was set to determine the treatment and control groups based on the criteria of whether or not the city was selected as a demonstration city for the green credit policy, with the treatment group being 1 and the rest of the cities being 0.

#### 3.3.3 Control variables

In order to control the other factors affecting residents’ health, this paper introduces a series of municipal-level control variables: (1) industrial soot and dust emissions (*smoke*), which is expressed as industrial soot and dust emissions; (2) industrial sulfur dioxide emissions (*so2*), expressed as industrial sulfur dioxide emissions in the forms of logarithmic terms; (3) fiscal spending capacity of local governments (*pfe*), is replaced by local general budget expenditures; (4) education level (*edu*), expressed as local general budget expenditures on education; (5) scale of the population (*pop*), which is substituted by the city’s average annual population count; (6) average wage of residents (*income*) represented by the average wage of employees in the workforce. For the purpose of avoiding bias such as heteroskedasticity due to excessive numerical difference, the control variables in this paper are all processed logarithmically.

### 3.4 Results of descriptive statistics

Descriptive statistics of the variables are shown in Table 1. Green credit policy has been enacted in 52.30% of the cities in the sample, and there is a large difference in the degree of health of the population between different regions (standard deviation of 2.67).

**Table 1:** Descriptive statistics of main variables.

Variable	Obs	Mean	Std	Min	Max
Rod	3,144	2.2788	2.6741	0.08	41.2023
Income	3,144	4.1503	0.5257	1.0413	6.7133
Lnso2	3,144	4.2564	0.5878	0.3010	5.7579
Lnpfe	3,144	6.4560	0.3376	5.0860	7.9258
Lnedu	3,144	5.6938	0.3445	4.1811	7.0598
Lnpop	3,144	2.5359	0.3168	1.3055	3.5327
Lnincome	3,144	4.7447	0.1684	4.2696	5.3042

## 4 Influence of green credit policy on residents’ health

### 4.1 Results of DID

Table 2 presents the results of the empirical regressions of the green credit policy effects on residents’ health. Column (1) controls for only individual and year fixed effects, with an interaction coefficient of −0.313, which passes the 1% significance test; Columns (2) to (7) sequentially add control variables to column (1), and after controlling for other influencing factors, the estimated coefficients of the model’s core explanatory variable (*DID*), are all significantly negative, suggesting that residents’ health is significantly improved in demonstration cities compared to non-demonstration cities. It reflects the fact that the green credit policy can effectively enhance residents’ health (Equation 1).

**Table 2:** Regression results of green credit policy on residents’ health.

Variable	(1)	(2)	(3)	(4)	(5)	(6)	(7)
DID	−0.313^***^ (0.115)	−0.316^***^ (0.116)	−0.320^***^ (0.115)	−0.350^***^ (0.113)	−0.348^***^ (0.115)	−0.350^***^ (0.113)	−0.350^***^ (0.110)
Lnsmoke		−0.041 (0.107)	−0.088 (0.113)	−0.062 (0.112)	−0.062 (0.112)	−0.009 (0.110)	−0.021 (0.107)
Lnso2			0.439^***^ (0.133)	0.334^**^ (0.131)	0.334^**^ (0.131)	0.258^**^ (0.129)	0.093 (0.126)
Lnpfe				−5.128^***^ (0.508)	−5.167^***^ (0.668)	−5.077^***^ (0.655)	−4.140 (0.643)
Lnedu					−0.058 (0.654)	−1.961^***^ (0.669)	−0.166 (0.673)
Lnpop						14.790^***^ (1.386)	12.398^***^ (1.363)
Lnincome							−11.665^***^ (0.931)
Con	2.326^***^ (0.033)	2.156^***^ (0.443)	0.822 (0.599)	−31.938^***^ (3.301)	−31.859^***^ (3.421)	−5.167 (4.185)	−47.845^***^ (5.311)
Obs	3,144	3,144	3,144	3,144	3,144	3,144	3,144
*R* ^2^	0.686	0.686	0.688	0.698	0.698	0.710	0.725
Id-fixed	Yes	Yes	Yes	Yes	Yes	Yes	Yes
Year-fixed	Yes	Yes	Yes	Yes	Yes	Yes	Yes

***, **, and * represent statistical significance at the 1, 5, and 10% levels, respectively. The values in parentheses are robust standard errors for clustering to the city level.

### 4.2 Parallel trend test

**Figure 3:**
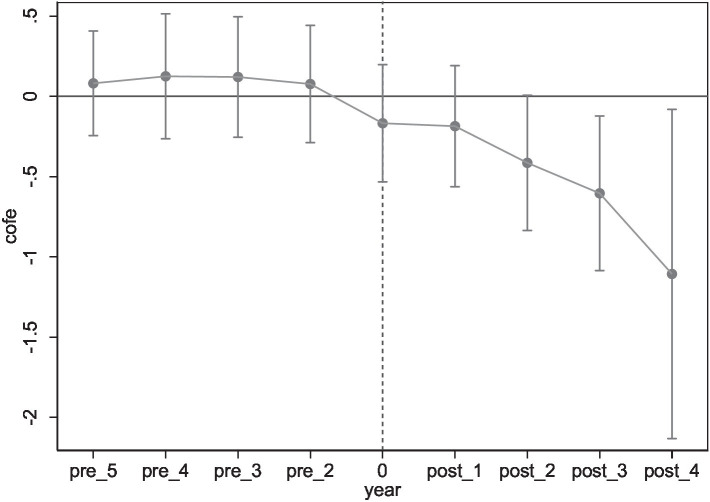
Parallel trend test.

In order to verify the dynamic effect of policy on residents’ health and examine whether it is a time-staggered exogenous shock, this paper adopts a parallel trends test, drawing upon the method established by Zhou et al. (37). Based on the exact year of implementation of the demonstration city, the sample interval is from the first 5 years before the policy was implemented (pre_5) to the year after the implementation (post_4). Where the fourth year of implementation (post_4) is an interval that includes the fourth year of implementation to the end year of the sample, and the year before the implementation (pre_1) is not included in the examination as the base year. From Figure 3, the pre-construction regression coefficients are not significant, indicating that there is no significant difference in residents’ health between the demonstration cities and the other control group cities. After the demonstration cities were constructed, the residents’ health in the demonstration cities was significantly improved, in essence, the green credit policy improved residents’ health, and this significant effect continued to increase during the demonstration period. Thus proving that the parallel trend hypothesis is satisfied between the treatment group and the control group.

### 4.3 Robustness tests

#### 4.3.1 Excluding other policy effects

Green credit policy was introduced in various regions mainly between 2017 and 2019, which is the same period during which other green economy policies were intensively introduced. Therefore, this paper excludes the effect of other environmental policies approach to this period. First, setting aside the effect of *the Environmental Protection Law of the People’s Republic of China*, which mandates rigorous controls on the discharge of pollutants in urban areas, this regulation potentially strengthens the limitations of industrial and municipal emission outputs. Second, the impact of excluding pilot green finance reform zones. In 2017, the Chinese government decided to set up “green financial reform and innovation pilot zones” in eight selected locations across five provinces in the country. The policy fully exerts the function of the financial market to support green financing. According to the previous analysis (38), the green credit policy improves residents’ health by optimizing the urban environment and enhancing the green financing function. If the pilot phase of a demonstration city is influenced by intersecting and concurrent policies, the baseline regression coefficients may encounter challenges in accurately reflecting the actual effects derived from the establishment of the demonstration city. To verify whether the above situation exists, dummy variables for the two policies, environmental protection law, and green financial reform pilot zone are added to the baseline regression equation. The results of the regression are presented in column (1) of Table 3, and the coefficient of the interaction term is −0.305 and is significant at the 10% level, indicating that the green credit policy helps to improve residents’ health.

**Table 3:** Robustness analysis.

Variable	(1)	(2)	(3)	(4)	(5)
DID	−0.305^*^ (0.184)	−0.262^***^ (0.084)	−0.262^***^ (0.082)	−0.148^***^ (0.049)	−0.262^***^ (0.082)
DID2	−11.491 (1.799)	–	–	–	–
DID3	−1.121 (0.943)	–	–	–	–
*R* ^2^	0.686	0.788	0.788	0.860	0.788
Controls	Yes	Yes	Yes	Yes	Yes
Id-fixed	Yes	Yes	Yes	Yes	Yes
Year-fixed	Yes	Yes	Yes	Yes	Yes
Id*year-fixed	Yes	Yes	Yes	Yes	Yes

Same as Table 2.

#### 4.3.2 Non-randomized selection problem

The selection of demonstration cities is influenced by factors such as the city’s geographic region, economic level, and resource endowment, and is not completely random so that inherent differences at the individual and temporal levels have different impacts on residents’ health. To mitigate the issue of self-selection bias, an interaction term between the individual and time-trend terms was added to the baseline regression in an attempt to capture the effect of individual variables over time on residents’ health and to mitigate estimation bias due to non-random selection as much as possible. The regression results are shown in column (2) of Table 3, where the *DID* coefficient is −0.262, which is significant at the 1% level, after adding the interaction terms of the individual and time trend terms. This suggests that the baseline regression is not affected by sample selection bias and the results are robust.

#### 4.3.3 Shrinkage treatment

With the aim of excluding the interference of extreme outliers, the paper applies the upper and lower tailing treatment at 1 and 5%, the results of which are shown in columns (3) and (4) of Table 3. The coefficients of the policy dummy variables after the shrinkage treatment are still significantly negative, which is consistent with the results of the baseline regression.

#### 4.3.4 PSM-DID test

In the baseline regression analysis, selecting demonstration cities as the treatment group for the difference-in-differences regression may lead to bias in regression results due to the non-randomness of sample selection. To further test the robustness of the above results, this paper utilizes the PSM-DID method to correct for possible bias due to sample selection. To avoid loss of sample size after matching, the control variable in the baseline regression was selected as the matching variable, and the samples of the treatment and control groups were matched year by year by the nearest-neighbor matching method based on the propensity score matching results. The tests of differences in the means of the matched control variables between the treatment and control groups are all non-significant, they satisfy the conditions of the test of balance in nature. To ensure the matching quality of the sample data, after obtaining the propensity scores a density functional plot was further plotted to check the common support domain after matching. The propensity scores of the demonstration cities’ participation sample and non-participation sample have a large range of overlap and most of the observations are within the common range of values. Using the matched samples of control variables to run difference-in-differences regressions again, the regression results for the PSM-DID model are obtained as shown in column (5) of Table 3. It can be seen that the coefficient of the dummy variable for green credit policy on residents’ health remains significantly negative, which is largely consistent with the results of the baseline regression, thus indicating the robustness of the conclusions obtained in the previous paper.

### 4.4 Placebo test

**Figure 4:**
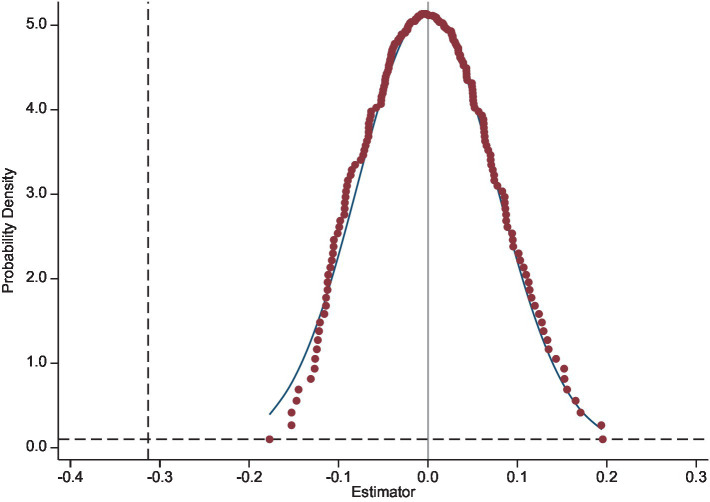
Placebo test.

Aiming to exclude other unobserved variables from interfering with the research conclusion, this paper conducted a placebo test by randomly assigning the treatment groups to the entire sample through random sampling. Referred to Hu et al. (39) method, this operation was repeated 200 times to improve the validity of the test. Figure 4 shows the scatter distribution of the probability density distribution of the coefficient estimates. The coefficient estimates for the placebo test were clustered around value of 0, most of which were not significant. The estimates from the baseline regression are not included in the test results. This suggests that the conclusions of this research were influenced by other unobserved variables.

## 5 Analysis of the mechanisms

The findings of this study indicate that the enactment of the green credit policy has significantly enhanced the health levels of residents. Therefore, the question arises: through what mechanisms does the enforcement of the policy contribute to the enhancement of residents’ health? In response, this research will explore and evaluate the mechanisms through which the green credit policy affects the improvements of the environment and the elevation of public services standards in demonstration cities.

### 5.1 Environmental improvement in demonstration cities

Column (1) of Table 4 examines the impact of the implementation of the green credit policy on the pollution emissions. The level of urban pollution emissions is measured by taking the natural logarithm of regional industrial effluent discharges (in tons). The regression results show that the coefficient of the interaction term in column (1) is −0.043 at the 5% significance level, indicating that the construction of the demonstration city reduces the level of pollution emissions in the city. The coefficient of the interaction term in column (2) is 0.543 at the 1% significance level, indicating that the level of urban pollution emissions has a significant effect on residents’ mortality, in essence, the green credit policy improves the urban environment by lowering the level of urban pollution emissions, which in turn can improve residents’ health. Hypothesis 1 is verified.

**Table 4:** Construction of demonstration cities and environmental optimisation.

Variable	Environmental improvement effects		Public services enhancement effect	
	Ww (1)	Rod (2)	Psas (3)	Rod (4)
DID	−0.043^**^ (0.020)		0.025^***^ (0.005)	
Ww		0.543^***^ (0.087)		
Pasa				−5.262^***^ (0.419)
Obs	3,072	3,072	3,072	3,072
R^2^	0.751	0.465	0.710	0.733
Controls	Yes	Yes	Yes	Yes
Id-fixed	Yes	Yes	Yes	Yes
Year-fixed	Yes	Yes	Yes	Yes

Same as Table 2.

### 5.2 Improved public services in demonstration cities

The green credit policy requires demonstration cities to develop intensive livelihood services, while at the same time providing local governments with the fiscal space to increase the supply of public services by alleviating fiscal pressures. Column (3) of Table 4 examines the impact of the implementation of green credit policy on the local public services standards. Indicators such as education and health care, which comprise the number of public services *per capita* at the city level, were used to reflect the number of public services *per capita* in the city as measured by principal component analysis. The regression results show that the construction of demonstration cities significantly improves the level of urban public services, and the regression coefficient of the interaction term is positive at the 1% significant level. Column (4) of Table 4 examines the effect of the level of public services on residents’ health, and the coefficient of the interaction term is −5.262 at the 1% level of significance, which suggests that an increase in the level of public services and facilities can significantly reduce the mortality rate of the population and improve residents’ health. Public services facilities positively affect residents’ health by improving the human living environment, degree of education and accessibility to medical services. Hypothesis 2 is verified.

## 6 Expansive research: heterogeneity analysis of green credit policy

There is an imbalance in China’s regional economic development due to the number of resources, geographic location, historical development, and other factors. Cities in different locations or levels differ in terms of infrastructure, development conditions and other aspects, so the policy effects of treatment groups have heterogeneity. Based on the research and analysis, this paper further explores the possible heterogeneity of the effects of green credit policy on residents’ health in terms of urban locations and resource endowment.

### 6.1 Heterogeneity analysis of urban locations

This paper categorizes the entire sample geographically into Eastern, Central, and Western sub-samples based on urban locations and conducts regression analyses independently for each. The results are presented in columns (1)–(3) of Table 5. It is evident that the green credit policy does not have a significant effect on the residents’ health in the eastern region and the central cities, but it can significantly contribute to the improvement of residents’ health in the western region. This phenomenon may be attributable to the comparatively advanced stage of comprehensive development level in the central and eastern regions, specifically regarding the evolution of infrastructure construction and the enhancement of public services. In contrast, the western region is in the period of development, lacking in development foundation. Cities in the central and eastern regions benefit from their geographic position, which endows them with a greater wealth of human and material resources, and a more accessible health care framework. Therefore, the implementation of the green credit policy might not induce a significant impact in these regions, as evidenced by the negligible effect observed. Conversely, the western region is experiencing a phase of expedited socio-economic advancement. The implementation of the green credit policy cultivates the optimization and progression of the industrial paradigm, thereby exerting a more significant policy effect than that observed in the eastern and central regions.

**Table 5:** Heterogeneity analysis.

Variable	Urban location			Resource endowment	
	(1) Eastern	(2) Central	(3) Western	(4) Resource-based	(5) Non-resource-based
DID	0.164 (0.234)	0.203 (0.178)	−1.072^**^ (0.470)	−0.362^**^(0.154)	−0.193 (0.240)
Obs	3,144	3,144	3,144	3,144	3,144
R^2^	0.724	0.724	0.729	0.725	0.724
Controls	Yes	Yes	Yes	Yes	Yes
Id-fixed	Yes	Yes	Yes	Yes	Yes
Year-fixed	Yes	Yes	Yes	Yes	Yes

Same as Table 2.

### 6.2 Heterogeneity analysis of urban resource endowments

Resource-based cities provide important energy resource security and strategic support for China’s economic development (39). However, the serious historical legacy problems of resource-based cities, such as the crude development model, have placed them under pressure for transition and upgrading. Therefore, testing the heterogeneous effects of green credit policy on residents’ health in resource-based and non-resource-based cities can provide more policy recommendations for the promotion of regional green development strategy. The sample was divided into resource-based cities and non-resource-based cities based on the Chinese government’s criteria for dividing resource-based cities, and then, re-running the regression. The results of columns (4)–(5) in Table 5 show that the estimated coefficients of resource-based cities are significantly negative, while the coefficient of non-resource-based cities fail the significance test. In an overarching assessment, the green credit policy exerts a more significant effect on the health levels of residents in resource-based cities. On one hand, the depletion of resources in China’s resource-based cities is increasingly acute, presenting significant challenges for economic transformation and upgrading. The introduction of green credit policy has offered financial support for these cities, which secures a more salubrious and integrated urban progression. On the other hand, under the “dual carbon” goals, local governments of resource-based cities facing substantial pressure to transform economic development models are intensifying their embrace of new economies and paradigms. In other words, resource-based cities face a greater necessity for industrial transformation and upgrading, which continually heightens their reliance on green credit funds, thereby indirectly enhancing urban environmental amelioration. Therefore, from a comprehensive perspective, the green credit policy more vigorously advances the eco-friendly economic growth of cities reliant on resources and enhances the health standards of the residents.

## 7 Discussion

Green credit policy occupies a crucial position in China’s green finance system, serving as a vital source of funding for the green and low-carbon development of the real economy and holding significant implications for improving living environments and promoting public health. This study discovers that the implementation of green credit policy has significantly enhanced the health levels of residents in demonstration cities. This positive outcome can be attributed to several factors. Firstly, green credit policy reflects the principles of sustainable development, aiming to fundamentally restrict the financing of enterprises that prioritize economic growth at the expense of environmental performance. By strengthening the restrictions and constraints on heavily polluting, high-energy-consuming, and high-emission industries, this policy effectively curbs excessive investment in these sectors, limits pollution sources, and rectifies urban environmental pollution. Secondly, the green credit policy prioritizes support for green industries and projects, incentivizing enterprises to transition and upgrade towards greener practices, thereby cultivating a harmonious coexistence between humans and nature. However, the sustainable development of green credit currently faces challenges. The study indicates that the health levels of residents in demonstration cities have only increased by 0.35. This limited improvement could be attributed to several factors. Firstly, the green credit standard system remains inadequate, with inconsistencies existing between the standards set by the People’s Bank of China (PBC) and the China Banking and Insurance Regulatory Commission (CBIRC). This discrepancy poses difficulties for commercial banks in identifying green credit projects and formulating targeted financial support measures. Secondly, enterprises face high costs associated with green and low-carbon transformation. This transition necessitates substantial financial investments, often with no guarantee of immediate returns. The absence of mandatory constraints on carbon emissions in China further exacerbates the situation, as many enterprises, after weighing the costs of pollution control, often lack the motivation to proactively embrace transformation. Thirdly, the incentive mechanisms are insufficient. While national policies encourage local governments to introduce supporting policies to facilitate the green and low-carbon transformation of enterprises, the actual implementation of these policies is often hindered by factors such as the level of economic development and local resource endowments. Therefore, some incentive policies prove difficult to implement, making it challenging to realize the full potential of green credit and thereby undermining enterprises’ motivation to invest in transformation.

Through an analysis of underlying mechanisms, this study indicates that demonstration cities primarily enhance residents’ health outcomes by enhancing environmental quality and elevating the provision of public services. Green credit policy, in conjunction with measures to reduce pollutant emissions and cultivate the adoption of clean energy sources, can effectively reduce hazardous substance concentrations, thereby augmenting air quality. Such improvements contribute to a reduction in the incidence of respiratory illnesses, strengthen the overall immune response among residents, and reduce the risks associated with allergies and other health complications. The enhancement of public services is primarily reflected in the expansion of green spaces and the beautification of urban landscapes, creating a more aesthetically pleasing and agreeable living environment, finally cultivating a greater sense of well-being and improved mental health among residents.

Heterogeneity analysis indicates that western regions and resource-based cities exhibit greater sensitivity to green credit policy. This phenomenon can be attributed to several factors. Firstly, western regions, often characterized by relatively lagging economic development and underdeveloped capital markets, experience suboptimal resource allocation. Green credit policy, accordingly, can function as a potent instrument to channel increased financial support, thereby motivating the implementation of green projects and cultivating sustainable development. Through the deployment of green credit, western regions can facilitate industrial structural upgrades, optimize resource allocation, and realize both economic and environmental benefits. Resource-based cities, in contrast to their non-resource-based counterparts, exhibit reliance on the extraction and utilization of natural resources for economic sustenance. This dependence frequently engenders environmental pollution and ecological degradation. Green credit policy, by implementing differentiated credit strategies targeting environmentally responsible enterprises and traditional, less sustainable businesses, plays a critical role in directing capital flows toward the former category and associated green projects. Accordingly, this facilitates economic transformation and sustainable development in resource-based cities. Moreover, green credit policy can offer an impetus for these cities to bolster environmental governance, ameliorate environmental quality, and enhance the overall quality of life for their residents.

In contrast to prior research, this study leverages panel data from demonstration cities to shed light on the relationship between green credit policy and residents’ health, thus broadening the scope of green credit policy effect research. By thoroughly accounting for heterogeneity among demonstration cities, including differences in the timing of green credit policy implementation, this study employs a multi-period difference-in-differences methodology to enhance result accuracy.

## 8 Conclusion and implications

Leveraging on the quasi-natural experiment of green credit policy, this paper examines the impact of green credit policy on the health of the population based on data of 262 prefectural cities in China from 2010 to 2021, using the difference-in-differences model (DID). The paper found that the green credit policy significantly improved residents’ health. Mechanism analyses show that the green credit policy positively affects residents’ health mainly through the improvements of the environment and the elevation of public services standards. Heterogeneity analyses show that the green credit policy has a more significant effect on improving residents’ health in the western region than those in the central and eastern regions, and resource-based cities are better than non-resource-based cities, which indirectly indicates that there is an uneven degree of demand for green credit products in different regions. The green credit policy eases financial pressure through social funding, frees up more resources for public services expenditures, and affects public health by improving environmental quality.

Considering the aforementioned conclusions and in alignment with China’s specific context, this study has derived the following insights and offers the following recommendations: Firstly, there should be support for enterprises’ green production to cultivate the advancement of green innovation technologies. Green credit policy, by directing funds from high-pollution industries to clean and environmentally friendly sectors, can achieve carbon emission reductions. It is necessary to strengthen the effect of “forced effect” of green credit policy on enterprises limited by green credits. This could be facilitated through measures such as reduced interest rates and subsidies for green innovation (rewarding achievements in green innovation), thereby encouraging heavily polluting enterprises to engage in green research and development, particularly those innovations in green technology that yield long-term carbon emission reduction effects. Secondly, there is a need to advance the enhancement of urban public services levels. The increasing of environmental regulation and the equalization of public services are protracted and profound processes that necessitate the gradual implementation of policies. This policy should incentivize local governments to promote green construction in a more efficient and sustainable way, thus enhancing resident well-being. Thirdly, the spatially uneven characteristics of the effect of green credit policy on residents’ health must be acknowledged. Support policies should be adapted to local conditions. A moderate increase in general transfer payments and policy preferences for the western region and resource-based cities would effectively advance regional green credit policy.

## 9 Limitations and future prospects

This research is not without several limitations as follows:

Firstly, the employed research methodology may lack the sufficient level of scientific rigor. The data collection and analysis process might be susceptible to unaccounted confounding factors and variables, potentially compromising the accuracy and reliability of the findings. Secondly, the presence of missing values in the sample data, despite employing imputation methods, could raise concerns regarding the accuracy and credibility of this study. Thirdly, the exploratory phase of green credit policy presents a significant limitation, as there is a dearth of well-established indicators to draw upon. Future research should aim to explore the multifaceted influences of green credit policy, employing a more comprehensive suite of indicators for a more robust and comprehensive measure of resident health outcomes.

## Data availability statement

The raw data supporting the conclusions of this article will be made available by the authors, without undue reservation.

## Author contributions

MW: Data curation, Formal analysis, Software, Writing – original draft, Writing – review & editing. YW: Conceptualization, Validation, Writing – original draft. BG: Supervision, Writing – review & editing.
